# Cardiovascular implications in adolescent and young adult hypertension

**DOI:** 10.31083/j.rcm2305166

**Published:** 2022-05-07

**Authors:** Rupesh Raina, Amrit Khooblall, Raghav Shah, Nina Vijayvargiya, Prajit Khooblall, Bhavya Sharma, Nikhil Datla, Aarushi Narang, Keval Yerigeri, Manasa Melachuri, Kirsten Kusumi

**Affiliations:** ^1^Akron Nephrology Associates/Cleveland Clinic Akron General Medical Center, Akron, OH 44307, USA; ^2^Department of Nephrology, Akron Children’s Hospital, Akron, OH 44308, USA; ^3^Department of Medicine, Northeast Ohio Medical University, Rootstown, OH 44272, USA; ^4^Department of Pediatric Nephrology, Akron Children's Hospital, Akron, OH 44308, USA

**Keywords:** Pediatric hypertension, Cardiovascular outcomes, Adolescent hypertension

## Abstract

**Background:**

Hypertension is one of the most prevalent diseases in the United States, affecting an estimated 3.5% of children and adolescents. It can be adversely affect most organ systems but is particularly detrimental to the heart and vascular systems. The repercussions can be gauged through well-established measures of cardiovascular function including left ventricular mass index (LVMI), left ventricular hypertrophy (LVH), carotid intima media thickness (cIMT), and aortic stiffness. Cardiovascular function is also affected by underlying etiologies of hypertension including chronic kidney disease, polycystic kidney disease, coarctation of the aorta, adrenal disorders, renal artery stenosis, obstructive sleep apnea, as well as various drugs and medications (decongestants, stimulants, Non-steroidal Anti-inflammatory Drugs (NSAIDs), and steroids).

**Methods:**

An exhaustive literature search was conducted for clinical data regarding pediatric hypertension. Sixty-seven articles were incorporated with data on 189,477 subjects total. The data was then extracted and categorized as relating to hypertension incidence, LVMI, LVH, cIMT, and/or aortic stiffness.

**Results:**

The prevalence of pediatric (<18 years) hypertension extracted from 47 studies from 1994 to 2018 averaged 4%. The LVMI assessed over 7 studies (n = 661) averaged 39.3 g/$m^{2.7}$ in the hypertensive cohort and 30.1 g/$m^{2.7}$ in the control cohort. The cIMT assessed over 7 studies (n = 580) averaged 0.55 mm in the hypertensive cohort and 0.49 mm in the control cohort. Ambulatory arterial stiffness parameters assessed over 5 studies (n = 573) in the normotensive cohort averaged 99.73 mmHg, 69.81 mmHg, 76.85 mmHg, and 46.90 mmHg, for SBP, DBP, MAP, and PP respectively. Ambulatory arterial stiffness parameters assessed over 5 studies (n = 573) in the hypertensive cohort averaged 129.56 mmHg, 73.69 mmHg, 95.08 mmHg, and 56.80 mmHg, for SBP, DBP, MAP, and PP respectively.

**Conclusions:**

The significance of pediatric hypertension is emphasized by evidence of early cardiovascular disease as demonstrated by non-invasive measures including cIMT and arterial stiffness parameters, and target organ damage and including LVH and LVMI factors. Thus, early diagnosis and treatment of high blood pressure is paramount for improving long term cardiovascular health and preventing long term morbidity and mortality.

## 1. Introduction

Hypertension (HTN) is one of the most prevalent diseases in the United States and is estimated to affect 34% of adults as well as 3.5% of children and adolescents [1, 2, 3]. According to the 2018 American Academy of Family Physicians guidelines, standard systolic blood pressure (BP) is defined as 90–110 mmHg and diastolic BP is 55–75 mmHg, whereas the mark for hypertension in children older than 13 is >130 mmHg and >80 mmHg, respectively. Children twelve and younger may deviate from this measure based on sex, age, height, and race weighted on a normative distribution with HTN classified as the 95th percentile. HTN can be categorized into several stages to assess the extent at which the disease has progressed: Stage 1, Stage 2, and hypertensive crisis. Stage 1 (BP levels 130–139/80–89 mmHg) is characterized by the presence of at least a singular cardiovascular (CV) risk factor and/or structural change in the heart and arteries without organ damage. Stage 2 (BP levels >140/90 mmHg) is characterized by persistent functional and structural changes in the heart, multiple cardiovascular markers, and indications of preliminary organ damage [1, 2, 3]. Patients in hypertensive crisis (BP levels >180/120 mmHg) suffer severe organ damage and are at risk of imminent CV and neurological events leading to high rates of morbidity and mortality [1, 2, 3]. The general progression of HTN drives Cardiovascular disease (CVD) and target organ damage.

Hypertension can be categorized as either primary (essential) or secondary. Primary hypertension is defined as chronic elevation in blood BP without a specified cause. In 90–95% of cases, modifiable and nonmodifiable risk factors play a major role in its development. Risk factors in pediatric patients with primary hypertension include maternal smoking, family history of hypertension, sedentary lifestyle, overweight/obesity, poor diet, unmanaged stress, male sex, and low birth weight [1, 2, 3, 4]. Secondary hypertension, present in 5–10% of cases, is defined as an elevation in BP due to a specific cause or structural disorders such as chronic kidney disease, polycystic kidney disease, coarctation of the aorta, adrenal disorders, renal artery stenosis, obstructive sleep apnea, and as a result of a number of drugs and medication [5]. The World Heart Foundation has identified early diagnosis and treatment of high BP, especially in children and adolescents, vital to improving cardiovascular health and preventing long term morbidity and mortality [6]. Left untreated, sustained hypertension can result in earlier onset of CVD, kidney dysfunction and/or end organ damage. This literature review assesses current data on the incidence of pediatric and adolescent hypertension, associated cardiovascular parameters, as well as the effects of secondary hypertension on cardiovascular parameters.

## 2. Methodology 

### 2.1 Data search

A literature search utilizing CINAHL (1980–2021), Cochrane (1980–2021), Medline/PubMed (1986–2021), and Web of Science (1965–2021) was conducted. Keywords included “pediatrics” AND “hypertension”, “blood pressure”, “LVH”, “LVMI”, “cIMT”, “AASI”, “PWV”, and “PP”. No restriction on time or geographic location was used. The search was further narrowed to only include relevant studies in the English language. A total of 28,645 articles were found from the initial search.

### 2.2 Data selection

Screening strategies are shown in the Patient/Population/Problem, Intervention/exposure, Comparison, and Outcome chart, or PICOS (**Supplementary Table 1**). The studies were included if primary data on a hypertensive pediatric cohort was reported. Animal studies, systematic reviews, and abstract-only literature that did not assess the significance hypertension in pediatric patients were excluded. After removing duplicate studies and excluding studies that did not meet the inclusion criteria, a total of 67 studies remained. **Supplementary Fig. 1** details a flow chart with the inclusion and exclusion citations created following the Preferred Reporting Items for Systematic Reviews and Meta-Analyses (PRISMA) guidelines.

### 2.3 Data extraction 

Data extraction was conducted by two independent individuals via full article examinations. If the studies were eligible, the sample sizes or individual cases of the patient populations were pooled together with respect to the hypertensive parameter category. From the included studies, the extracted data included number of subjects, age, sex, study design, study geography, and specified cardiovascular parameters. These results were compared with the reference ranges observed in the healthy subjects. All the analyses were performed using Microsoft Excel.

## 3. Etiology

### 3.1 Incidence

Our meta-analysis evaluates the incidence of pediatric and adolescent hypertension from 1994–2018 over 46 studies (n = 187,663) and has demonstrated a stable frequency of 4% (Range: 1–13%) (Table 1) [7, 8, 9, 10, 11, 12, 13, 14, 15, 16, 17, 18, 19, 20, 21, 22, 23, 24, 25, 26, 27, 28, 29, 30, 31, 32, 33, 34, 35, 36, 37, 38, 39, 40, 41, 42, 43, 44, 45, 46, 47, 48, 49, 50, 51, 52, 53]. Song *et al*.’s [54] systematic review over 47 articles (n = 94,675) demonstrated that the prevalence of childhood hypertension has increased from approximately 75% to 79% among children between ages 6 to 19 years from 2000 to 2015. This rapid increase is a cause for concern as children and adolescents with hypertension are prone to developing lifelong cardiovascular disease [49, 50, 51, 52, 53, 54].

**Table 1.** **The incidence of pediatric (<18 years) hypertension extracted from 47 studies from 1994 to 2018**.

Last name	Year	Sample sizes	Cases	Age range	Frequency
Verma M, *et al*. [7]	1994	2560	28	5–15	1%
Adrogue HE, *et al*. [8]	2001	14686	147	10–15	1%
Sorof JM, *et al*. [9]	2002	2460	70	12–16	3%
Iman S, *et al*. [10]	2003	2910	198	5–13	7%
Rezende DF, *et al*. [11]	2003	607	15	7–14	2%
Sorof JM, *et al*. [12]	2004	5102	221	10.3–19.4	4%
Subhi MD, *et al*. [13]	2006	1427	25	6–12	2%
Ataei N, *et al*. [14]	2007	6038	48	13–18	1%
Chiolero A, *et al*. [15]	2007	5207	191	10–14	4%
McNiece KL, *et al*. [16]	2007	6790	638	11–17	9%
Ostrowska-NL, *et al*. [17]	2007	25309	1240	7–18	5%
Savitha MR, *et al*. [18]	2007	503	31	10–16	6%
Taksande A, *et al*. [19]	2008	2643	152	6–17	6%
Moore WE, *et al*. [20]	2009	1829	42	5–17	2%
Stergiou GS, *et al*. [21]	2009	765	14	6.1–17.9	2%
Katona E, *et al*. [22]	2011	10213	258	15–18	3%
Leung LC, *et al*. [23]	2011	6093	88	6–18	1%
Wang R, *et al*. [24]	2011	1140	46	6–14	4%
Steinthorsdottir SD, *et al*. [25]	2011	970	30	4–14	3%
Acosta AA, *et al*. [26]	2012	1010	25	15.4	2%
Rinaldi AEM, *et al*. [27]	2012	903	29	6–14	3%
Hong B, *et al*. [28]	2012	4175	400	11–17	10%
Kumar J, *et al*. [29]	2012	990	34	10–19	3%
Xu YJ, *et al*. & Zheng YS, *et al*. [30, 31]	2012	2438	138	7–14	6%
Cinteza E, *et al*. [32]	2013	4866	358	3–17	7%
Ujunwa FA, *et al*. [33]	2013	2694	146	10–19	5%
Basiratnia M, *et al*. [34]	2013	2000	236	11–17	12%
Meng L, *et al*. [35]	2013	6304	197	3–18	3%
Baradol RV, *et al*. [36]	2014	2800	33	10–16	1%
Outdili Z, *et al*. [37]	2014	5207	113	10.1–14.9	2%
Patil RR, *et al*. [38]	2014	958	29	6–16	3%
Bloetzer C, *et al*. [39]	2015	5207	113	10–14	2%
Menghetti E, *et al*. [40]	2015	2007	124	6–17	6%
Derezinski T, *et al*. [41]	2015	416	51	14	12%
Saury-Paredes LA, *et al*. [42]	2016	259	16	5–11	6%
Zhang X, *et al*. [43]	2016	7781	465	6–18	6%
Badeli H, *et al*. [44]	2016	2072	144	7–17	7%
de Oliveira L, *et al*. [45]	2017	481	31	14–19	6%
Okpokowuruk FS, *et al*. [46]	2017	200	7	3–17	4%
Ajayi IO, *et al*. [47]	2017	1760	226	3–17	13%
Bloetzer C, *et al*. [48]	2017	5207	113	10–14	2%
Cheung EL, *et al*. [49]	2017	21062	569	10–19	3%
Krzywinska-WM, *et al*. [50]	2017	4941	435	10–18	9%
Balsara SL, *et al*. [51]	2018	2094	146	10–19	7%
Deren K, *et al*. [52]	2018	1024	17	12–17	2%
Rodrigues PR, *et al*. [53]	2018	1555	58	6–9	4%
**Total/Average**		**187663**	**7735**		**4%**

### 3.2 Risk factors

Risk factors associated with hypertension can be divided into non-modifiable and modifiable. Nonmodifiable risk factors include age, family history, genetic predisposition, sex, race and ethnicity. BP increases with age and most adults develop hypertension by the age of 70 [55, 56, 57, 58, 59, 60]. Modifiable risk factors include obesity, diabetes mellitus, diet, physical activity, alcohol, and tobacco use, all known risks to children and adolescents. Poor dietary habits including high salt intake and low potassium intake are also correlated with elevated BP in children and adolescents [61, 62, 63].

The relationship between BP and CVD applies primarily to systolic BP, especially in children and adolescents [64, 65]. BP has proven to be a major risk factor for subsequent CVD development independent of other CVD risk factors [65]. Left unchecked, sustained hypertension can damage the cardiovascular system and kidneys, including left ventricular mass(LVM), carotid intima media thickness (cIMT), aortic arterial stiffness (AAS), pulse wave velocity (PWV), peripheral vascular disease (PVD). This review identifies these events/outcomes and analyzes their relationship with elevated BP as well as the correlation between hypertension and CVD.

## 4. Pathophysiology

Cardiac output is the product of stroke volume (SV) (volume of blood pumped by each heart beat) and heart beats per minute. SV is dependent on the force of contraction and resistance in the vascular system. BP is the product of the cardiac output and peripheral resistance. Consequently, inadequate volume regulation, enhanced vasoconstriction and changes in the arterial wall (including increased resistance and/or decreased luminal diameter) can contribute to hypertension [66, 67]. This balance in arterial tone is also affected by intravascular volume and neurohumoral systems. Maintenance of homeostatic BP levels involves a complex interplay of various elements of an integrated neurohumoral system such as the renin-angiotensin-aldosterone system (RAAS), natriuretic peptides, sympathetic nervous system (SNS) and the immune system [68].

An elevated cardiac output is more commonly seen in the pediatric population while increased vascular resistance and vascular stiffness is more prevalent in hypertensive adults [66, 67, 68, 69, 70, 71]. Increased vascular stiffness can be attributed to increased α-adrenoceptor stimulation and/or the increased release of angiotensin and endothelins, which hasten the process of vascular remodeling [67, 71]. Aging and increasing vascular stiffness also augment pulse pressure and escalate afterload on the left ventricle [65, 66, 70]. Higher cytosolic calcium levels in the smooth muscle vasculature promotes sustained vasoconstriction in hypertensive patients. Consequently, an increase in both vascular stiffness and vascular resistance leads to increased cardiac load on the left ventricle (LV), resulting in left ventricular hypertrophy (LVH) and left ventricular diastolic dysfunction [67, 71].

In contrast, high blood pressure in children is often due to an underlying secondary cause. Thus evaluation of pediatric hypertension is more comprehensive and includes comorbidities, risk factors, and evidence of target organ damage [72, 73]. Renal parenchymal abnormalities account for roughly 75% of secondary hypertension in children, followed by renovascular abnormalities. Therapeutic and illicit drugs (e.g., corticosteroids, decongestants) as well as stimulants like caffeine or attention deficit disorder medications can be iatrogenic contributors [73]. Less common causes include pediatric tumors (Wilms tumor, neuroblastoma, and pheochromocytoma), Williams syndrome, Turner syndrome, endocrinopathies (e.g., hypercortisolism, hyperaldosteronism, and diabetes), congenital adrenal hyperplasia, and systemic lupus erythematosus [72, 73].

## 5. Parameters

### 5.1 Left ventricular mass index and hypertrophy

**Fig. 1.**
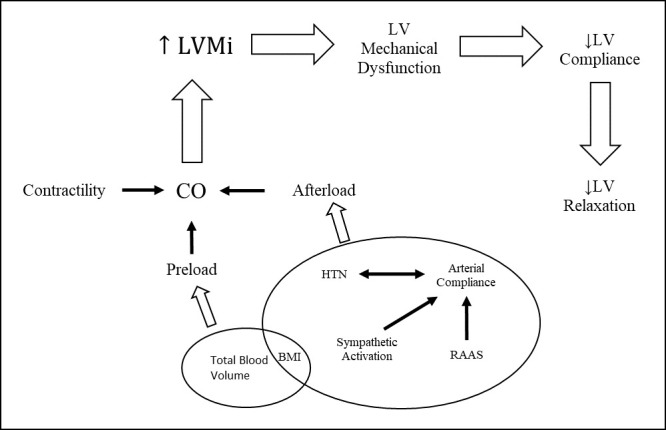
**Mechanisms associated with development of LVH and Diastolic Dysfunction**. Left ventricle remodeling due to hypertension leads to hypertrophy of cardiomyocytes with eventual fibrosis and diastolic dysfunction.

Left ventricular (LV) mass is a meausure of weight of the LV and includes the additive effect of BP on the heart. Increased blood pressure raises the afterload and requires the cardiac tissue to produce more force. To compensate the heart undergoes hypertrophy increasing in size and mass. Thus, primary hypertension among children and adolescents is often associated with the sequela of LV hypertrophy (LVH) and accordingly left ventricular mass index (LVMI) [74]. LVH is a compensatory reaction which starts as physiologi-cal but may become pathological; large increases in LVMI indicate target organ damage so it is often used as a surrogate outcome for cardiovascular risk in children and adolescents [75, 76]. LVMI is calculated using echocardiography and/or cardiac MRI and is used to quantify hypertension-induced LVH. An increased risk in cardiac mortality is associated with an LVMI of >51 g/$m^{2.7}$ for the adult hypertensive population, but such a predictive cutoff has not been established for children [75, 76]. Fig. 1 highlights the mechanisms associated with left ventricle dysfunction. Left ventricle remodeling due to hypertension is mediated by the interaction of cardiomyocytes and non-myocytes such as endothelial cells and fibroblasts [77]. Mechanical stretch activates intracellular signaling cascades leading to gene expression and synthesis of sarcomere proteins. The stress on the LV chamber is reduced by increasing the size of the cardiomyocyte (by adding sarcomeres in parallel if there is a pressure overload or in series if there is a volume overload) [78]. The mechanical stretch and upregulation of inflammation can trigger fibroblasts to differentiate into myofibroblasts, causing myocardial fibrosis. This impairs cardiac contraction or filling leading to heart failure with preserved or reduced ejection fraction and cardiac arrhythmias [79, 80, 81].

The strain of a hypertensive LV can be visualized using echocardiography by measuring the relative wall thickness and mass. This can be compared to the wall thickness and mass of a normotensive cohort. Our meta-analysis over seven studies (n = 661) observed a statistically significant increase (*p *< 0.05) in LVMI in hypertensive pediatric patients compared to controls at 39.3 g/$m^{2.7}$ vs. 30.1 g/$m^{2.7}$ (Table 2) [82, 83, 84, 85, 86, 87, 88]. The consistently increased LV mass can be attributed to pressure overload due to diastolic dysfunction.

**Table 2.** **Left ventricular mass index (LVMI) values extracted from 7 studies of pediatric/adolescent cohorts**.

Last name	Year	Age	Sample size	LVMI (g/$m^{2.7}$)	Control
Mirchandani *et al*. [82]	2014	8–18	109	42.3	-
Mir *et al*. [83]	2016		35	32.9	28.8
Meng *et al*. [84]	2015	9–15	232	34	28 ± 6
Lande *et al*. [85]	2006	12–17	35	36	-
Sorof *et al*. [86]	2003	11–16	32	46.8	31.4
Bjelakovic *et al*. [87]	2015	10–16	94	46.6	38.2 ± 8.8
Stabouli *et al*. [88]	2009	5–18	124	36.8	29.5 ± 8.3
**Total/Average**			**661**	**39.3**	**30.1**

Several studies have shown LV diastolic dysfunction in children with primary arterial hypertension can be measured via tissue Doppler imaging (TDI) [89]. Zamojska *et al*. [89] evaluated systolic and diastolic function in children (n = 64) and found that the LV myocardial workload (based on echocardiographic evaluation with the use of standard and tissue Doppler echocardiographic parameters) was higher in children with hypertension (0.46 ± 0.08 vs. 0.35 ± 0.03; *p *< 0.01). The value of the A wave was higher in hypertensive children (0.59 ± 0.12 m/s vs. 0.49 ± 0.09 m/s; *p *< 0.01), indicating distended venous pressure [89]. The velocity of mitral flow propagation was lower (0.61 ± 0.08 m/s vs. 0.72 ± 0.10 m/s; *p *< 0.01) and E/Vp ratio (LV filling) was higher (1.50 ± 0.27 vs. 1.21 ± 0.23; *p *< 0.01) in hypertensive children [89]. Isovolumetric relaxation and deceleration times were significantly higher in patients with BP elevation, thus compensating for an increased demand and increasing cardiac workload [89].

Stabouli *et al*. [88] assessed the LVM in normotensive, prehypertensive and hypertensive children and adolescents (n = 124). They demonstrated that LVMI in the prehypertensive cohort was significantly higher (29.5 ± 8.3 g/$m^{2.7}$) (*p *< 0.05, Mann-Whitney Test) that those of the normotensive cohort. This emphasizes the importance of preventing children from even becoming prehypertensive as well as consistently monitoring at-risk children for modifiable and nonmodifiable risk factors. Additionally, Lee *et al*. [90] observed that pediatric hypertensive subjects had significantly higher LVMI’s than the control group (*p* = 0.297). This highlights the importance of consistent monitoring of LVMI in addition to LVH.

### 5.2 Carotid intima-media thickness

The intima and media are the innermost layers of the artery and are prone to mechanical and physiologic dysfunction due to intravascular or extravascular forces. High BP can directly induce the carotid wall’s hypertrophy (intimal/medial thickening) as hemodynamic factors (e.g., local distending pressure, pulsatile load, and shear stress) induce intrinsic changes in the arterial walls. The carotid artery intima-media thickness is often evaluated in hypertensive individuals to gauge amassed endothelial deposits [90, 91, 92]. The presence or progression of endothelial deposition is more commonly referred to as atherosclerosis and its onset can begin as early in childhood or adolescence. Fatty streaks have been observed in the aorta, coronary and/or carotid arteries of patients as young as 2 years old [91].

Our meta-analysis of seven studies (n = 580) found a statistically significant increase in cIMT in hypertensive children and adolescents compared to normotensive controls (0.55 mm vs. 0.49 mm, *p *< 0.05) (Table 3) [83, 84, 85, 86, 93, 94, 95]. The increased cIMT measurements in these patients can be attributed to structural and functional changes in the carotid artery.

**Table 3.** **Carotid intima-media thickness (cIMT) values extracted from 7 studies of pediatric/adolescent cohorts**.

Last name	Year	Age	Sample size	cIMT (mm)	Control
Mir *et al*. [83]	2016	<18	35	0.46	0.35
Meng *et al*. [84]	2015	9–15	232	0.49	0.46
Litwin *et al*. [93]	2004	6–20	110	0.45	0.41
Gil *et al*. [94]	2008	13–18	32	0.62	0.5
Lande *et al*. [85]	2006	12–17	35	0.67	0.63
Sorof *et al*. [86]	2003	11–16	32	0.72	0.63
Antoniewicz *et al*. [95]	2006	5–20	104	0.47	0.43
**Total/Average**			**580**	**0.55**	**0.49**

Litwin *et al*. [93] demonstrated mechanical dysfunction in the arterial wall (e.g., distensibility) in hypertensive children. The cross-sectional compliance (change in volume to change in pressure) and β-stiffness coefficient were greatly increased in the hypertensive cohort (594 ± 154 ${\mathrm{mm}}^{2}$/mmHg and 4.11 ± 0.80) compared to the normotensive cohort (410 ± 176 ${\mathrm{mm}}^{2}$/mmHg and 3.8 ± 0.8) (*p* = 0.003 and N/A) [93]. The β stiffness index is a measure of arterial stiffness that is not dependent on the blood pressure at the time of recording, rather it is solely determined by the characteristics of the exact location of the artery. The cross-sectional area of the wall was increased 22.4 ± 44.0 ${\mathrm{mm}}^{2}$ in the hypertensive cohort compared to 17.9 ± 4.1 ${\mathrm{mm}}^{2}$ in the normotensive cohort (*p* = 0.0001) [93]. Compliance is related to the diameter of the artery; hence, significantly greater diameter values in hypertensive patients leads to more compliant vessels. Conversely, the cross-sectional distensibility (arterial expansion and contraction) was significantly decreased in the hypertensive cohort (41.3 ± 9.9 kPa*${\mathrm{10}}^{-1}$ ${\mathrm{10}}^{-3}$) compared to the normotensive cohort (48.4 ± 10 kPa*${\mathrm{10}}^{-1}$ ${\mathrm{10}}^{-3}$) [93]. This reflects the shifts in the elastic/collagenous properties of the arterial wall. Gil *et al*. [94] observed similar shifts in elastic property as cross-sectional compliance (0.15 ± 0.04 ${\mathrm{mm}}^{2}$/mmHg vs. 0.23 ± 0.10 ${\mathrm{mm}}^{2}$/mmHg) and distensibility (0.0053 ± 0.0021 ${\mathrm{mmHg}}^{-1}$/${\mathrm{10}}^{-2}$ vs. 0.0087 ± 0.0045 ${\mathrm{mmHg}}^{-1}$/${\mathrm{10}}^{-2}$) of the carotid artery were also significantly different between hypertensive and normotensive group (*p *< 0.05).

### 5.3 Aortic stiffness (AASI, PWV, PP)

Arterial stiffness can result from arteriosclerosis and/or atheromatosis. Arteriosclerosis occurs when there is an increase in the rigidity and thickness of the arterial wall often leading to hypertension. Atheromatosis is a change in arterial inflammation, which leads to increased lipid deposition in the arterial walls and endothelial dysfunction [96]. The less compliant central vasculature changes hemodynamic flow and arterial pressure, thereby influencing cardiac function and coronary perfusion. Arterial stiffness plays a significant role in the development of CVD in the elderly population, and its role in the pediatric population is rapidly evolving, particularly in children with obesity [96]. The development of minimally invasive measurements of arterial stiffness, such as ambulatory arterial stiffness index (AASI), pulse wave velocity (PWV) and pulse pressure (PP), has been beneficial in diagnosing and predicting CVD.

AASI measures arterial stiffness via ambulatory BP monitoring (ABPM) during 24 hours of normal activity, providing prognostic information on cardiovascular mortality and target organ damage [97, 98, 99]. Arterial stiffness has a nonlinear relationship to distending pressure where an increase in mean arterial pressure is associated with an exponential increase in arterial stiffness. In individuals with elastic arteries, systolic and diastolic BPs vary according to changes in mean arterial pressure such that systolic and diastolic BPs rise with activity and fall during rest. However, individuals with stiff, inelastic vasculature are less able to respond to changes in physiologic requirements [97]. Our meta-analysis on pediatric AASI over 5 studies (n = 573) demonstrates elevated average 24-hour systolic BP and diastolic BP in hypertensive vs normotensive children at 129.56 mmHg vs. 99.73 mmHg and 73.69 mmHg vs. 69.81 mmHg, respectively (Table 4) [21, 100, 101, 102, 103]. Simonetti *et al*. [100] assessed a hypertensive pediatric cohort (n = 114) and reported higher AASI values in hypertensive children compared to normotensive participants (0.370 ± 0.120 vs. 0.204 ± 0.199, *p *< 0.0001) [Odds Ratio: 8.2 (95% CI 4.2–16.2)]. AASI in all participants was also statistically correlated to SBP (*$r^{2}$* = 0.0363, *p* = 0.0096) and 24-hour SBP (*$r^{2}$* = 0.0363, *p* = 0.0096) and inversely correlated with 24-hour DBP (*$r^{2}$* = 0.09657, *p* = 0.0008), reinforcing its distension of vasculature in hypertension [100]. Stergiou *et al*. [21] found similar statistical correlations, but went on to note positive associations in bivariate coefficients between AASI and LVM (*r* = 0.37), LVMI (*r* = 0.24) and stroke volume (*r* = 0.17). Table 5 (Ref. [9, 85, 86, 104, 105]) shows a more comprehensive review of the association of of AASI with cardiovascular outcomes such as LVMI, LVM and cIMT. A positive correlation was also found regarding the amount of time since diagnosis with hypertension and AASI values. Specifically, pediatric participants suffering from chronic kidney disease for >3 years had higher AASI values than those whose condition was diagnosed within 3 years (0.378 ± 0.106 vs. 0.311 ± 0.109, *p* = 0.014) [100]. In conclusion, the longer the duration of hypertensive disease, the greater the vascular damage due to decreased compliance in diastolic BP.

**Table 4.** **Ambulatory arterial stiffness index measurements, pulse velocity, and pulse pressure data points extracted from 5 studies of pediatric/adolescent cohorts**.

		N	Years	24-hour (mmHg)			
		Sample	Age	SBP	DBP	MAP	PP
Stergiou *et al*. [21]	Normotensive	66	12.8	115.10	64.70	-	50.40
	Hypertensive	16	14	135.60	74.30	-	61.30
Simonetti *et al*. [100]	Normotensive	71	12.1	109.50	66.40	80.70	43.40
	Hypertensive	114	12	125.50	77.90	93.70	47.60
Kollios *et al*. [101]	Normotensive	45	10.09	108.78	64.42	95.64	-
	Hypertensive	10	11.2	123.70	71.30	102.33	-
Skrzypczyk *et al*. [102]	Normotensive	-	-	-	-	-	-
	Hypertensive	177	15.04	128.17	70.84	89.95	57.53
Skrzypczyk *et al*. [103]	Normotensive	20	15.55	119.85	65.55	83.70	54.20
	Hypertensive	54	15.12	134.85	74.11	94.33	60.78
**Total/Average**	**Normotensive**	**202**	**12.635**	**99.73**	**69.81**	**76.85**	**46.90**
	**Hypertensive**	**371**	**13.472**	**129.56**	**73.69**	**95.08**	**56.80**

Abbreviations: SBP, Systolic blood pressure; DBP, Diastolic blood pressure; MAP, Mean arterial pressure; PP, Pulse pressure.

**Table 5.** **Univariate analyses between anthropomorphic and ambulatory arterial stiff ness index variables to cardiovascular outcomes such as LVMI, LVM, and cIMT**.

Univariate analyses between outcome and potential predictors						
Last Name; Year	Sample Size	Age	Outcome	Predictor	r	*p*-value
Sorof *et al*. 2002 [9]	37	13.5 ± 3.7	LVMI	Ambulatory SBP index	0.43	0.008
				24-hour SBP	0.34	0.037
				24-hour SBP load	0.38	0.02
				Daytime SBP load	0.37	0.025
				Nighttime SBP	0.33	0.048
				Nightime SBP load	0.38	0.021
Sorof *et al*. 2003 [86]	32	13.9 ± 2.7		Weight	0.42	0.02
				BMI	0.49	0.005
Urbina *et al*. 1995 [104]	160	9–22		SBP	0.21–0.27	0.05
Daniels *et al*. 1995 [105]	201	6–17	LVM	Age	0.72	-
				Height	0.81	-
				Weight	0.84	-
				Body Surface Area	0.87	-
				Lean body mass	0.86	-
				Fat Mass	0.54	-
				SBP	0.58	-
				DBP	0.48	-
Sorof *et al*. 2003 [86]	32	13.9 ± 2.7		Weight	0.5	0.003
				BMI	0.43	0.014
				LVMI	0.54	0.001
				Interventricular septal thickness	0.58	0.001
				Posterior wall thickness	0.54	0.001
Lande *et al*. 2006 [85]	35	12–17	cIMT	24-h SBP load	0.51	0.009
				24-h DBP load	0.5	0.01
				Daytime mean SBP	0.43	0.003
				Nighttime DBP index	0.4	0.04
				Daytime systolic load	0.54	0.005
				Daytime diastolic load	0.56	0.004
				Daytime diastolic index	0.54	0.005
				Daytime mean diastolic index	0.54	0.005
				Daytime mean DBP	0.54	0.005
Sorof *et al*. 2002 [9]	37	13.5 ± 3.7	Interventricular Septal Thickness	24-hour SBP	0.43	0.008
				Wake SBP load	0.44	0.007
				Sleep SBP	0.39	0.017
			Left Ventricular Posterior Wall	24-hour SBP	0.41	0.012
				Wake SBP load	0.39	0.016
				Sleep SBP	0.41	0.012

Abbreviations: LVMI, Left ventricular mass index; LVM, Left ventricular mass; cIMT, carotid-intima media thickness.

Pulse Wave Velocity (PWV) measures the speed at which pressure waves travel through blood vessels. Changes in the vessel’s ability to dilate, particularly as the arterial wall becomes stiffer, increase the rate at which the waves travel through the circulatory system. The increased rate of arterial pressure causes an increase in the rate of the reflected wave back to the right atrium. To compensate, ventricular systolic pressure increases [106]. An increase in the elastic modulus, vessel wall thickness and blood density with a decrease in vessel radius is associated with a higher PWV.

Due to variations in the segmental lengths at which pulse wave velocity can be measured, direct comparisons amongst multiple pediatric/adolescent studies are limited. Individual studies have shown statistically significant differences in PWV between hypertensive and normotensive cohorts. Stergiou *et al*. [21] observed an increased PWV (7.4 ± 1.2 m/s vs. 6.3 ± 1.7 m/s, *p* = 0.02) between a hypertensive and normotensive cohort, respectively. Mir *et al*. [83] (5.87 ± 0.87 m/s vs. 5.29 ± 0.67 m/s, *p* = 0.02), Meng *et al*. [84] (5.31 ± 1.44 m/s vs. 5.08 ± 8.0 m/s), and Gil *et al*. [94] (7.459 ± 0.632 m/s and 7.592 ± 0.608 m/s vs. 6.906 ± 0.560 m/s and 7.026 ± 0.515 m/s, respectively; *p *< 0.05) all observed similar trends despite varying methods in calculating PWV. Such a consistent association between PWV as a marker of arterial stiffness validates its role as a preclinical measure of cardiovascular disease.

Pulse pressure (PP) is determined by the direct force of ventricular ejection and the viscoelasticity of arteries and can be calculated as SBP-DBP. It can also be calculated indirectly by wave reflections [107]. Wave reflections are formed when forward-moving blood is partially reflected back from arterial obstructions. Increased arterial stiffness causes the vessel’s wave reflections to become larger and arrive earlier in the systole, increasing PP [108]. Endothelial stress and arteriosclerosis increase PP as loss of vascular compliance increases SBP (while simultaneously decreasing DBP) [108]. Arteriosclerosis increases arterial stiffness, driving hypertension and increases in PP. Elevated PP has been associated with adverse cardiovascular outcomes and death, hence PP is a rough estimate to gauging arterial stiffness in relation to cardiovascular complications [109]. Overall, measures of aortic stiffness including AASI, PWV, and PP are all limited by the lack of pediatric references based on height, weight, age, and ethnicity. There is also a lack of standardization in AASI and PWV measurements and variation in the equipment used [107, 108, 109].

Our meta-analysis of PP over 5 studies (n = 573) compares the value between hypertensive and normotensive children at 56.80 mmHg vs. 46.90 mmHg, respectively (Table 4) [21, 100, 101, 102, 103]. Stergiou *et al*. [21] noted positive associations in bivariate coefficients between PP and 24-hour AASI (*r* = 0.50), LVM (*r* = 0.68). LVMI (*r* = 0.33), PWV (*r* = 0.36), stroke volume (*r* = 0.36) and peripheral resistance (*r* = 0.12). Simonetti *et al*. [100] also found consistent increases between 24-hour PP and AASI (*$r^{2}$* = 0.1341, *p *< 0.0001). This highlights PP’s intertwined relationship to aortic stiffness as well as other cardiovascular parameters (e.g., LVH and cIMT).

## 6. Discussion

Secondary hypertension is common in pediatric patients with studies reporting a prevalence of 75–85% [110, 111, 112, 113]. The underlying causes typically vary with age; coarctation of the aorta and renal disorders are more common in children up to 6 years old whereas renal parenchymal disease is more likely to affect those between 6 to 10 years old [114]. Pediatric patients are typically referred to subspecialists for a comprehensive evaluation of secondary causes of hypertension, however, rising cases of obesity and essential hypertension are becoming more common. The 2020 update of the American Academy of Pediatrics (AAP) Recommendations for Preventative Pediatric Health Care suggests that young children with stage 1 or stage 2 hypertension as well as those with difficult to treat hypertension should be evaluated for secondary causes of hypertension [115]. More importantly, causes of secondary hypertension in children who are overweight, have stage 1 hypertension, or otherwise healthy children with new-onset hypertension should not be ruled out prematurely [116].

### 6.1 Chronic kidney disease

**Fig. 2.**
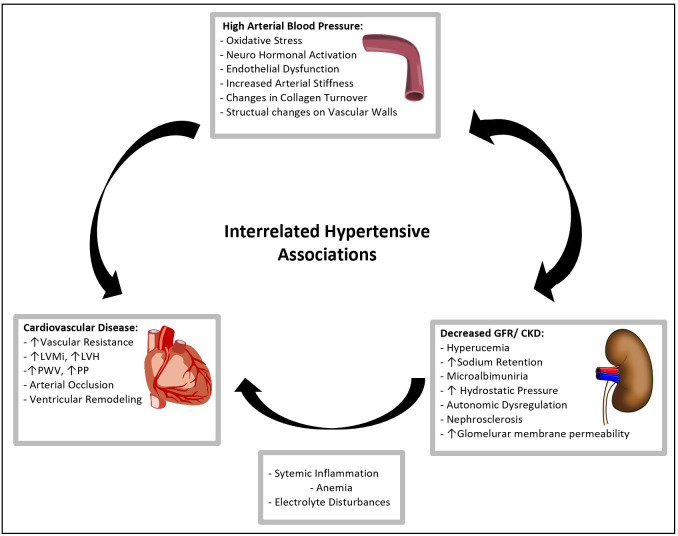
**Interrelated Hypertensive Associations**. A representation of the linked mechanisms of Hypertension, CKD, and CVD. Elevated blood pressure accelerates the interaction of endothelial dysfunction, oxidative stress, vascular atrophy and RAAS dysregulation which exacerbates the progression of cardiovascular disease.

**Fig. 3.**
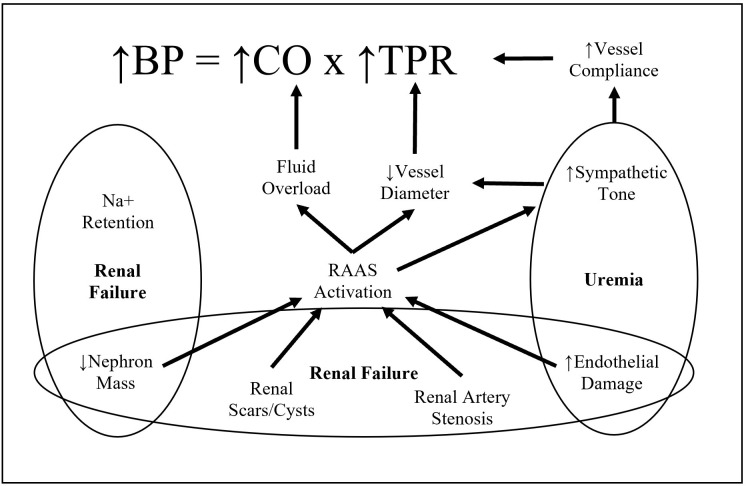
**Factors associated in hypertension due to chronic kidney disease**. A representation of the interdependent mechanisms of CKD and Hypertension. CKD, characterized by renal failure and uremia results in sympathetic overactivity of RAAS and eventual increase of cardiac output and total peripheral resistance.

Chronic kidney disease (CKD) and hypertension are deeply interdependent pathophysiologic states as sustained hypertension can worsen kidney function, and, loss of kidney function can drive hypertension (Fig. 2). Sympathetic overactivity, RAAS dysregulation, salt retention and volume overload are all directly affected by and mediate both disorders [117] (Fig. 3).

CKD is associated with sympathetic overactivity, which stimulates RAAS, reducing blood flow in the peritubular capillaries downstream of sclerosed glomeruli. Glomeruli then increase renin secretion which increases circulating angiotensin II levels, driving increased systemic vascular resistance and BP [118]. Since there are fewer functioning glomeruli in CKD, the remaining glomeruli hyperfilter, increasing systemic arterial pressure and pulse pressure [118]. The overall net loss of GFR reduces sodium excretion rates, while angiotensin II also promotes sodium reabsorption in the proximal tubule and collecting duct. This consequently leads to an increase in extracellular volume, vasoconstriction and increased peripheral resistance, which the body attempts to counteract with release of the natriuretic hormone.

This association between progression of CKD and hypertension is emphasized by Raina *et al*.’s [119] statistical analyses of a crosssectional pediatric CKD cohort (n = 620). They demonstrated a significant positive correlation between PP and LVMI ($R^{2}$ = 0.005; *p* =0.029) as well as an inverse correlation between PP and GFR ($R^{2}$ = 0.017; *p* = 0.011) indicating the association of loss of GFR with loss of vascular compliance and developing CVD.

These associations are also supported by Reynolds *et al*. [120] who demonstrated that non-glomerular CKD progression risk increases 3-fold and glomerular CKD progression risk increases 2-fold if time-varying systolic BP supersedes the 90th percentile in pediatric patients. Dionne *et al*. [121] analyzed longitudinal ambulatory BP in a pediatric cohort (n = 679) with CKD via mean arterial pressure (MAP). In children with non-glomerular CKD, MAP >the 90th percentile was associated with disease progression risk after 4 years (HR, 1.88 [CI, 1.03–3.44]). In those with glomerular CKD, the risk for progression was present from baseline with the highest risk in those with MAP ¿90th percentile (HR, 3.23 [CI, 1.34–7.79]) [121]. The earlier onset of CKD progression in glomerular CKD may be linked to greater inflammation. Effective management of hypertension in CKD is vital to reducingtherateatwhichpatientsprogresstoend-stage renal disease. The lifespan of a pediatric or adolescent patient can be severely reduced due to CKD-associated CVD and ESKD [122, 123]. Chavers *et al*. [122] observed a pediatric chronic dialysis cohort (n = 1454) and found that 31.1% (n = 452) developed cardiac-related events such as valvular disease (11.7%), cardiomyopathy (9.6%) and cardiac arrest (3%). These events were notably increased in adolescents (15–19 years old) (*p *< 0.0001). Of the 7% (n = 107) who died, 38% (n = 41) were a result of cardiac deaths. Parekh observed a similar pediatric cohort (n = 1380) and found that the cardiac deaths affected 22.5% (n = 311) [123].

### 6.2 Polycystic kidney disease

Polycystic kidney disease is one of the most com-mon inherited kidney diseases [124]. It is caused by mu-tation in either PKD1 or PKD2 genes, of which PKD1 ac-counts for 85% of the cases [125].

Enlarging renal cysts can cause abdominal pain, hypertension, recurrent urinary tract infections (UTI) and renal stones. Common extrarenal manifestations include polycystic liver disease, in-tracranial aneurysms, valvular heart defects and abdominal hernias [126]. Patients with PKD generally develop symptoms in the adult age group [124], but this disease can manifest at any age [127]. Moreover, Very Early Onset (VEO) PKD (diagnosis in-utero or within 18 months of delivery) is associated with worse clinical outcomes such as development of early onset hypertension,CVD and ultimately End Stage Renal Disease (ESRD) [128].

Hypertension is a prominent feature of Autosomal dominant polycystic kidney disease (ADPKD) and is seen in the pediatric population. Marlais *et al*. [129] observed that the prevalence of hypertension in children with ADPKD is 20%, whereas, prevalence in the general is estimated to affect 3–5% pediatric population [130]. Various mechanisms have been proposed for the development of hypertension in ADPKD. In particular, the activation of RAAS by the enlarging of renal cysts is believed to play a major role [131]. Additionally, activation of RAAS and the favoring intrarenal ischaemia affects the sodium sensitivity and activity of the SNS which is known to augment over stimulation of the SNS in ADPKD patients and accelerate the onset of hypertension [132].

The most common cardiovascular outcome associated with PKD are cardiac hypertrophy and cardiac remodeling [132]. An increase in the left ventricular wall thickness is frequently detected in ADPKD patients and has a significant correlation with borderline (blood pressure 75–95th percentile) and high (>95th percentile) blood pressures (*p <* 0.0005 and *p *< 0.02) [133]. Chinali *et al*. [134] demonstrated that the prevalence of abnormal LV geometry was significantly higher in ARPKD vs. controls (33 vs. 0%; *p *< 0.005). They also concluded that increased relative wall thickness was more prevalent in ARPKD pediatric patients compared to healthy children (RWT = 0.35 ± 0.1 vs. 0.27 ± 0.03; both *p *< 0.001) [134].

This data illustrates the increased prevalence of hypertension in pediatric patients with PKD and the increased risk of CVD in PKD patients. Furthermore, hypertension is common in these patients even before the onset of renal insufficiency. Effective hypertension treatment is a key factor determining patient morbidity and mortality, and early aggressive therapeutic intervention to mitigate the progression of CVD is paramount for improving long term outcomes.

### 6.3 Coarctation of the aorta

Coarctation of the aorta is the fifth most common congenital heart defect with an incidence of 1 in 2500 live births [135]. It involves a narrowing of the aortic isthmus, often with subsequent tubular hypoplasia. The etiology is currently unknown and likely multifactorial; familial cases with associated gene deletions have been reported [136], but and evidence-based unifying theory has yet to be outlined.

Narrowing of the aorta in coarctation increases the pressure load on the proximal vessel in comparison to the aorta distal of the isthmus. The heightened mechanical stress induces rapid gene expression for collagen production, leading to remodeling of the vessel and increased resistance to pressure-induced dilatation [137]. Stiffening of the vessel contributes to an isolated systolic hypertension (with increased pulse pressure) similar to what is seen in vascular calcinosis. This hypertensive effect disseminates throughout the upper vasculature and commonly presents in infants as a drastic blood pressure difference between the upper and lower extremities.

Sezer *et al*. [135] investigated the effects of stenting procedure on left ventricular function, aortic stiffness, elasticity and systemic hypertension in children with coarctation of the aorta and found that the enduring cardiovascular effects can not always be reverted. The elasticity of the ascending aorta was found to be lower (6.4 ± 3.4 vs. 10.0 ± 1.7 ${\mathrm{cm}}^{2}$ ${\mathrm{dyn}}^{-1}$${\mathrm{10}}^{-6}$) and the aortic stiffness was found to be higher (5.6 ± 1.6 vs. 2.5 ± 0.45 ${\mathrm{cm}}^{2}$ ${\mathrm{dyn}}^{-1}$${\mathrm{10}}^{-6}$) compared with the control group even after endovascular stenting [138]. If left untreated, severe aortic narrowing may precipitate into heart failure or other cardiovascular complications such as aortic aneurysm. Rapid detection and treatment is critical for optimal outcomes, especially if narrowing progresses to closure of the arterial duct [139].

### 6.4 Adrenal disorders

There are several adrenal disorders which can cause hypertension in pediatric populations. These disorders can be split into dysfunction or hyperactivity of the adrenal cortex or the adrenal medulla. The prevalence of adrenal medulla disorders ranges between 1:2500 and 1:6500, of which 20% is of pediatric patients. The typical disorder of the adrenal medulla is pheochromocytoma, paragangliomas, or secretions of norepinephrine, epinephrine, dopamine and associated metabolites resulting in hypertension [140]. The disorders affecting the adrenal cortex and causing low renin hypertension include 1β-hydroxylase deficiency, alpha-hydroxylase deficiency, primary aldosteronism, familial hyperaldosteronism, and Cushing’s syndrome [141].

While norepinephrine and epinephrine induce hypertension by increasing endothelial contraction, aldosterone is a mineralocorticoid hormone involved with inflammation, vascular remodeling and oxidative stress resulting in organ damage, and derangement of fluid or electrolyte balances. Aldosterone drives salt retention, inflammation, and vasoconstriction as a protective response against dropping blood pressures. However, unregulated levels of aldosterone can cause endothelial damage by decreased nitric oxide synthase activity while increasing superoxide anion generation. These free radicals, in combination with the additional function of aldosterone retaining water/salt and inducing inflammation through transcription of nuclear factor-κB (NF-κB), interleukin 1 and 6, driving monocyte and macrophage infiltration. The subsequent inflammation and aldosterone mediated salt and water retention causes ischemic vascular, renal, and myocardial damage [142].

The systemic effects of aldosterone has been extensively studied in both adult and pediatric populations. A study using cardiac MRI imaging of 35 patients with primary hyperaldosteronism found decreased left ventricular ejection fraction compared to non-hypertensive controls (59.0 ± 7.3% vs. 62.1 ± 4.4%, *p* = 0.081) and increased left ventricular mass index compared to non-hypertensive controls (65.8 ± 16.5 g/$m^{2.7}$ vs. 44.1 ± 8.9 g/$m^{2.7}$ for HC; *p *< 0.001) [143]. These results demonstrate the discordant effects of prolonged elevation of aldosterone levels have on cardiac remodeling. Another study by Demirkiran *et al*. [144] demonstrated that subjects with primary aldosteronism had significantly lower flow-mediated dilation (3.3 [2.4–7.4] % vs. 14.7 [10.3–19.9] %, *p *< 0.01) and significantly higher cIMT (0.9 [0.7–1.0] mm vs. 0.8 [0.6–0.9] mm, *p* = 0.02) compared to patients with essential hypertension. These results indicate a loss of vascular compliance.

### 6.5 Renal artery stenosis

Renovascular HTN is elevation in BP due to the narrowing of renal blood vessels. It is the most common cause of secondary HTN, responsible for 5–25% of HTN in children [145]. Renovascular hypertension results in activation of RAAS secondary to reduced blood flow to part or all of one or both kidneys [146, 147]. The underlying mechanism of the various derivations of renovascular hypertension revolves around decreased perfusion to the kidney and activation of the RAAS pathway. Prolonged ischemia increases the amount of renin expressing cells in the kidney which ultimately raises blood pressure via vasoconstriction in the heart and kidney, sympathetic nervous stimulation, aldosterone stimulation, and fi-broblast stimulation (thus thickening the vascular wall, myocardium, and fibrosis) [147, 148]. Renovascular HTN can result from multiple disorders pathologies including renal artery stenosis, fibromuscular dysplasia (FMD), arteritides such as Takayasu arteritis (TA) or mid aortic syndrome, extrinsic compression of a renal artery, and renal artery infarction [146, 147, 148, 149]. Renovascular hypertension prevelence is related to geographic location where FMD is more commonly found in North America and Europe while TA is more prevalent in Asia and South Africa [150, 151].

Renovascular hypertension should be suspected when blood pressure control is refractory to hypertensive medication or when an abdominal bruit is observed [150, 151].

Lu *et al*. [151] assessed 14 pediatric patients diagnosed with renovascular hypertension and observed a mean blood pressure of 187/127 mmHg at the time of diagnosis. Congestive heart failure was found in a subset of the cohort which highlights the importance of measuring blood pressure in pediatric clinical practice. Compared to adults where 70–80% of patients have largely non-correctable atherosclerotic lesions, children with renovascular hypertension typically have correctable lesions [152].

### 6.6 Obstructive sleep apnea

Obstructive sleep apnea (OSA) affects approximately 1–5% of children and is characterized by partial or complete obstruction of the upper airway and subsequent disruption in sleep and/or proper gas exchange [153]. The sequalae from OSA in children can include failure to thrive, enuresis, behavioral problems, poor scholarly performance, and cardiopulmonary disease [154]. The correlation between pediatric OSA and secondary hypertension has been well documented and the pathophysiology with the most current literature will be discussed subsequently [155, 156, 157, 158, 159].

The most common etiology of obstruction in children with OSA is adenotonsillar hypertrophy [154]. However, the role of obesity, upper airway inflammation, neurological hypotonia, and craniofacial anatomy is also significant. Regardless of the cause, repeated nighttime obstructions may lead to intermittent hypoxia, hypercapnia, and sleep fragmentation [158]. These factors further lead to the observable clinical signs and symptoms including, snoring, frequent awakenings, witnessed apneic episodes, enuresis and cognitive/behavioral problems. Pulmonary and systemic hypertension is of specific importance in this population due to its large potential for morbidity and mortality in the setting of untreated OSA, Data has demonstrated a dose-specific relationship between severity of OSA and potential for secondary hypertension [160, 161]. Furthermore, LVH and heart failure have been reported in children with severe OSA [157, 162].

Li *et al*. [157] studied 306 children aged 6–13 years and found significantly higher awake and nocturnal BP values in those with OSA, when compared to healthy children. The severity of OSA was directly correlated with worsening of BP values, with moderate to severe OSA patients having an increased risk for nocturnal systolic (OR 3.9, 95% CI 1.4–10.5) and nocturnal diastolic (OR 3.3, 95% CI 1.4–8.1) values [157]. The end organ manifestations of hypertension secondary to OSA often appear cardiac in nature. In 2019, Hanlon *et al*. [162] analyzed 61 obese or overweight children with OSA and found 71.7% of this population to had clinically apparent LVH on echocardiography. Children with OSA also had a significantly increased risk of LVH (85.7% vs. 59.4%, *p* = 0.047). After adjusting for age, sex, race, and BMI, OSA was still associated with 4.11 times increased odds of displaying LVH on echocardiogram (95% CI 1.15–14.65; *p* = 0.030) [162]. Multiple other studies also document the associations between OSA and ventricular pathology [156, 157, 163, 164, 165].

Few studies have evaluated the effect of OSA treatment on cardiovascular parameters in children. Cincin *et al*. [165] studied myocardial performance and anatomy before and after adenotonsillectomy in 30 patients with diagnosed OSA. They demonstarted an improvement in both right ventricular (RV) (0.515 ± 0.066 vs. 0.434 ± 0.052, *p *< 0.0001) and LV (0.383 ± 0.079 vs. 0.316 ± 0.058, *p* = 0.018) performance indices following surgical treatment when compared to preoperative testing. Improvements in pulmonary artery pressure were also observed following adenotonsillectomy in this cohort [165]. A few other studies have found similar but not reproducible results [166, 167]. The data on treatment and prevention of hypertension and cardiovascular sequalae in children with OSA is preliminary, but promising. Further research on how to identify CVD in those with OSA early and prevent long term pathologic effects.

### 6.7 Medication/Drugs

NSAIDs are anti-inflammatory drugs with the potential of resulting in harmful side effects for the pediatric population [168]. Misurac *et al*. [169] reports a long-term study performed at their institution showing a 2.7% prevalence of NSAID induced AKI. The inhibition of prostaglandin production that occurs through NSAID use affects the kidney’s ability to modulate its GFR, especially when dehydrated. The resulting hypoperfusion of the glomerulus can result in medullary ischemic injury leading to acute renal failure. NSAIDs also have the propensity to increase sodium and water retention resulting in worsening of hypertension which could result in secondary complications such as heart failure and lower leg edema. Out of 1015 patients included in the study by Misurac *et al*. [169], 27 pediatric patients were diagnosed with NSAID-associated AKI. Further stratification of the data showed that 21 of those patients with NSAID-associated AKI had acute tubular necrosis either by clinical course or biopsy results. Additional research and randomized controlled trials are needed to full investigate the all-encompassing effects NSAID’s and stimulants in pediatric hypertension. With the high incidence of sinusitis in the pediatric population, the increased prescription of decongestants specifically targets the pediatric population [170]. The alpha-1 agonist activities of these decongestants can result in a dangerous side-effect of medication-induced hypertension which must be accounted for.

## 7. Lifestyle modifications

The importance of maintaining a healthy lifestyle is emphasized by the fact that confounding variables, most notable being obsese/overweight, may mask underlying causes of secondary hypertension. Obese and overweight body mass index (BMI) offen occur due to poor nutrition and lack of physical activity and have a significant influence on the onset of hypertension in pediatric patients and can augment cardiovascular implications [171].

The pathophysiology of hypertension in obesity and overweight children can be attributed to the hyperactivity of the sympathetic nervous system, and heart rate variability can serve an important non invasive marker. Heart rate variability can be categorized into low frequency (LF) indicating sympathovagal activity and high frequency (HF) indicating vagal activity which compose a normalized ratio called the sympathovagal balance [172]. A group of obese children and adolescents showed that the LF/HF ratio was significantly higher in obese children than controls which indicates heart sympathetic overactivity resulting in a higher systolic blood pressure [172]. Insulin resistance has also been implicated in the pathogenesis of secondary hypertension arising from obesity. Insulin resistance associated with obesity is known to inhibit glucose uptake but preserve the renal sodium retention effects causing a chronic volume overload and blood pressure elevation [171]. These mechanisms are associated with increased blood pressure and CVD including endothelial dysfunction, Left ventricular hypertrophy, and myocardial changes. Additionally, poor nutrition primarily characterized by excessive sodium intake can onset hypertension and cardiovascular outcomes. Sodium intake is more prevalent and detrimental to the progression of hypertension in obese children compared to healthy controls. Specifically, for every 1000 mg increase in sodium intake per day, the risk for elevated BP in obese children compared to normal weight children was 74% compared to 6%, respectively. Sodium intake is associated with increased risk of cardiovascular implications primarily CVD, stroke, and LVH [171, 173].

Obesity as a result of poor exercise and nutrition are detrimental risk factors that have a significant influence on the progression of CVD and cardiovascular implications. Berenson *et al*. [174] of the Bogalusa Heart Study observed a significant association between increased BMI and systolic blood pressure with fatty streaks and plaques in the coronary arteries (*r* = 0.60, *p *< 0.001). Sorof *et al*. [86] revealed that increased cIMT levels were also positively associated with BMI (*r* = 0.43) a key marker for endothelial dysfunction. LVH is also a primary adverse outcome of obesity associated hypertension and can lead to the progression of CVD. Bartkowiak *et al*. [175] observed that the prevalence of LVH in obese children vs. non-obese children was 14% compared to 3.6% respectively. LVMI was also greater in the obese children compared to non-obese children (36.1 ± 8.6 vs. 28.7 ± 6.9 g/$m^{2.7}$, *p *< 0.001), respectively. Furthermore, Hanevold *et al*. [176] delineated that the prevalence of LVH in patients with >95th percentile BMI for age was 41.1% (OR, 5.02, 95% CI 2.17–11.61, *p*-0.002).

Obesity and nutrition have significant effects on the progression of CVD. In addition, the prevalence and severity of obesity is drastically increasing in children. Encouraging fitness and quality nutrition in pediatric patients in addition to earlier screening for hypertension in patients with poor nutrition and fitness levels may be advantageous in managing the progression of CVD. Overall, current studies of long-term cardiovascular outcomes in children with hypertension are limited; however, there is growing evidence that childhood hypertension continues into and worsens throughout adulthood. In the Bogalusa Heart Study, children with increased BP were 2–3 times more likely to develop essential hypertension as young adults [177]. Similarly, Zhou *et al*. [178] found that in a 19.1 year follow up, untreated and uncontrolled hypertensive adults were at an increased risk for CVD (coronary artery disease, arrythmias, heart failure, heart valve dysfunction, heart attack, and stroke related mortalities) compared to patients who were normotensive or treated for hypertension. HTN not only affects coronary vessels but significantly affects cerebral and systemic vasculature. Thus, untreated hypertensive patients not only had a greater CVD specific mortality (HR = 1.77) compared to treated patients, but also had an increased cerebrovascular disease (cerebral vessel ischemia, stenosis, thrombosis, embolism, or hemorrhage) mortality (HR = 2.53) and all-cause mortality (hazard ratio = 1.40) [178]. The severity and progression of CVD, cerebrovascular disease, and all-cause mortality and morbidity due to untreated hypertension highlights the importance of maintaining BP homeostasis, especially in pediatric patients.

## 8. Conclusions

Sustained hypertension can lead to a number of adverse cardiovascular outcomes, all of which are accentuated in the pediatric population due to their longterm complications. Not only are functional parameters dysregulated in the cardiovascular system, but exponentiating target organ damage can also develop—especially in the renal system. Fortunately, these cardiovascular parameters including LVMI, LVH, cIMT, AASI, PWV, and PP provide noninvasive measures of early CVD. The well-established literature outlines the degree of correlation of each parameter and emphasizes its importance to cardiovascular health. Thus, prompt detection and intervention are imperative to prevent and control hypertension, especially in pediatric populations with often overlooked underlying conditions, to minimize the risk of longterm cardiovascular disease.

## Supplementary material

Supplementary material associated with this article can be found, in the online version, at https://doi.org/10.31083/j.rcm2305166.
