# Supplementary material for: Cardiovascular implications in adolescent and young adult hypertension
Source: Rev Cardiovasc Med. 2022 May 7;23(5):166. doi: 10.31083/j.rcm2305166 (PMC11273899; doi:10.31083/j.rcm2305166)
Supplement: Supplementary file 1 [file 2153-8174-23-5-166-s1.zip › 2153-8174-23-5-166-s1.doc]

Table 1. PICOS table to describe the inclusion and exclusion criteria for the systematic review.

| PICO elements | Inclusion Criteria | Exclusion Criteria | Search Terms |
| --- | --- | --- | --- |
| Population | All hypertensive pediatrics of age or sex. | n<1 | “pediatrics AND” (see below) |
| Intervention/  Prognostic Factor | Presence of pediatric hypertension | Reports only focusing on obesity |  |
| Comparison | Non-hypertensive pediatric patients |  |  |
| Outcomes | Any outcomes | Missing data (e.g. follow up data, demographics) |  |
| Questions | Is there a statistical difference between the cardiovascular parameters (LVH, LVMI, cIMT, AASI, PWV, and PP) between hypertensive and non- hypertensive pediatrics. |  | “pediatrics” AND “hypertension”, “blood pressure”, “LVH”, “LVMI”, “cIMT”, “AASI”, “PWV”, and “PP” |
| Study Types | Retrospective, Prospective, Clinical Trial, and Case Series Reviews | Case studies, Literature Reviews, Animal studies, Systematic Reviews. Abstract only |  |

**Identification**

Records identified from*:

CINAHL, EMBASE, Medline, PubMed, and Web of Science (n = 28,645)

Records removed *before screening*:

Duplicate records removed (n = 14,652)

**Screening**

Records screened

(n = 13,993)

Records excluded**

(n = 11,364)

Reports sought for retrieval

(n = 2,629)

Reports not retrieved

(n =342)

Reports assessed for eligibility

(n =2,287)

Full-text articles excluded, with reasons (n=1,163)

Wrong publication type:

(n=642)

Animal studies (n=412)

**Included**

Studies included in review

(n=67)

**Fig. 1. The Preferred Reporting Items for Systematic Reviews and Meta-Analyses or PRISMA flow shows the systematic categorization of studies in order to include the most pertinent studies for the data extraction.**
